# Gain or amplification of 1q21 in systemic light chain amyloidosis is associated with advanced Mayo stage, plasma cell disease and worse overall survival

**DOI:** 10.1007/s00277-025-06256-7

**Published:** 2025-03-22

**Authors:** Sara Oubari, Maria Papathanasiou, Lars Michel, Tienush Rassaf, Andreas Thimm, Tim Hagenacker, Daniela Ehling, Stefan Wieczorek, Eyad Naser, Ute Hegenbart, Stefan Schönland, Ulrich Dührsen, Hans Christian Reinhardt, Alexander Carpinteiro

**Affiliations:** 1 Department of Hematology and Stem Cell Transplantation, West German Cancer Center, University Hospital Essen, University Duisburg-Essen, Hufelandstraße 55, Essen, 45147 Germany; 2 Interdisciplinary Amyloidosis Network, University Hospital Essen, University Duisburg-Essen, Essen, Germany; 3 Department of Cardiology and Angiology, University Hospital Frankfurt, Frankfurt am Main, Germany; 4 Department of Cardiology and Vascular Medicine, West German Heart and Vascular Center, University Hospital Essen, University Duisburg-Essen, Essen, Germany; 5 Department of Neurology and Center for Translational Neuro- and Behavioral Science, University Hospital Essen, Essen, Germany; 6 Department of Medical Genetics, MVZ Dr. Eberhard & Partner Dortmund, Dortmund, Germany; 7 Institute of Molecular Biology, University of Duisburg-Essen, Essen, Germany; 8 Department of Internal Medicine V, Amyloidosis Center Heidelberg, University Hospital Heidelberg, Heidelberg, Germany; 9 German Cancer Consortium (DKTK), Partner Site University Hospital Essen, Essen, Germany

**Keywords:** AL amyloidosis, IFISH, +1q21, Mayo stage

## Abstract

Systemic light-chain amyloidosis (AL) is an acquired protein misfolding disease characterized by deposition of immunoglobulin light-chain fibrils most often secreted from clonal plasma cells. In this retrospective study we analyzed the impact of iFISH aberrations on clinical characteristics and outcomes in 175 AL patients presented between 2015 and 2024. The most common aberrations were t(11;14) (57%), deletion 13q14 (33%), +1q21 (21%), hyperdiploidy (21%) and deletion 16q23 (17%). Significant elevations in dFLC levels were observed in patients with + 1q21 (median 407 vs. 213 mg/l, *p* = 0.04) and deletion 16q23 (median 476 vs. 204, *p* = 0.006). Only + 1q21 was associated with increased levels of cardiac biomarkers NTproBNP (median 9945 vs. 3538 pg/ml, *p* = 0.002) and hsTnT (median 110 vs. 53 ng/l, *p* = 0.002). This resulted in an increased proportion of patients with Mayo stage IIIb (53% vs. 26%, *p* = 0.01). Patients with + 1q21 had more advanced plasma cell disease (*p* = 0.0004). Our study highlights for the first time + 1q21 as the key aberration associated with advanced cardiac and plasma cell disease. After 17 months of follow-up, overall survival was significantly worse in patients with + 1q21 treated with daratumumab (7.2 months vs. not reached, *p* = 0.006). Alternative therapeutic approaches such as CAR-T therapies or bispecific antibodies should be further investigated.

**Supplementary Information:**

The online version contains supplementary material available at 10.1007/s00277-025-06256-7.

## Introduction

Systemic light chain (AL) amyloidosis is a rare acquired protein misfolding disorder with an incidence of approximately 5–13 per million person-years [1]. It is characterized by extracellular deposition of misfolded amyloidogenic immunoglobulin light chain fibrils, most often secreted by clonal plasma cells in the bone marrow [2]. All organs except the central nervous system can be affected, most commonly the heart and kidneys [2, 3]. The outcome of patients with AL amyloidosis is strongly associated with the severity of organ involvement, particularly the heart. The Mayo classifications from 2004 to 2012 have been established to assess the severity of the disease [4–6].

Current treatment protocols are based on anti-plasma cell drugs [7, 8]. Treatment regimens have changed over time; while conventional or high-dose regimens with melphalan and perhaps dexamethasone were mainly used until 2010, bortezomib-containing regimens have been used since then in first-line setting [9]. In 2021, daratumumab, an IgGk monoclonal anti-CD38-antibody, was approved in combination with bortezomib, cyclophosphamide and dexamethasone for the first-line treatment of AL amyloidosis in Mayo stages I-IIIa [10].

Cytogenetic aberrations detected by interphase fluorescence in situ hybridization (iFISH) are well-described in multiple myeloma and have been incorporated into risk stratification systems; in the revised international staging system (R-ISS), high-risk was defined as the presence of at least one of the three high-risk aberrations: del (17p), t(4;14), or t(14;16). In the R2-ISS staging system, gain and/or amplification of chromosome 1q was further considered as independent poor prognostic factors [11, 12].

In AL amyloidosis, cytogenetic aberrations were described at an early stage [13, 14] and later further characterized with regard to their prognostic significance and influence on treatment response [15–23]. The t(11;14) translocation has been described as a negative factor for prognosis and treatment response in patients treated with bortezomib-containing regimens in the first-line setting [17, 19, 20]. In contrast, the presence of t(11;14) has been described as a positive prognostic factor for response to high-dose melphalan [18]. In a retrospective study of patients with recurrent or refractory AL amyloidosis treated with daratumumab, patients with gain 1q21 and/or hyperdiploidy had worse overall survival (OS) and shorter hematological event-free survival (hem-EFS) [24]. In contrast, patients with t(11;14) showed improved hem-EFS [24]. The Andromeda trial showed that the negative impact of t(11;14) regarding hematological response and major organ deterioration-event free survival could be reversed by adding daratumumab to bortezomib-containing chemotherapy [10, 21]. In daratumumab monotherapy, gain or amplification of 1q21 (+ 1q21) was reported to be associated with a reduced major organ deterioration progression-free survival and OS [22]. Recently, +1q21 was identified as an independent negative prognostic factor for hem-EFS [23].

In the present study we investigated the influence of iFISH aberrations on clinical characteristics and outcome in AL amyloidosis.

## Methods

This retrospective study included 175 patients with histologically confirmed AL amyloidosis who were presented at our center between April 2015 and August 2024 (Table 1). Ethical approval for this study was obtained from the ethics committee of the Medical Faculty, University of Duisburg-Essen, approval number 20–9458 BO and performed according to the Helsinki Declaration of 1975, as revised in 2013.

### iFISH-analysis

CD138-positive bone marrow plasma cells were isolated, and hybridization was performed using chromosome specific iFISH-probes. To avoid false positive results and clinically irrelevant sub-clonal aberrations, a threshold of 10% was applied for identifying gains, losses and translocations. The aberrations of interest were translocations t(11;14), t(4;14), t(14;16), t(14;20), deletions/monosomies 17p13, 13q14, 16q23, gains or amplifications of 1q21, and hyperdiploidy that was defined based on the score of Wuilleme et al. [25], which requires extra copies of at least two of the following three chromosomes: 5, 9, and 15. Hyperdiploidy and + 1q21 were quantified as the total percentage of aberrant cells [26].

## Plasma cell disease and organ involvement

The underlying plasma cell dyscrasia was diagnosed as recommended by the International Myeloma Working Group based on CRAB-criteria [27, 28]. The Freelite^®^ Test from Binding Site, Birmingham, United Kingdom, was used to measure free light chain levels. Organ involvement was assessed as recommended by the 10th International Symposium on Amyloid and Amyloidosis [29].

**Table 1:** Patient characteristics of the whole cohort (*n* = 175), 93 patients were treated in the daratumumab era and 68 in the pre-daratumumab era (either bortezomib-based regimens in 61 patients or other regimens in 7). Other regimens included five patients treated with melphalan and dexamethasone, one patient with high-dose melphalan directly and one patient with lenalidomide and dexamethasone

Patient characteristics	Whole cohort (*n* = 175) Number of patients (%)	Daratumumab era (*n* = 93) Number of patients (%)	Pre-daratumumab era (*n* = 68) Number of patients (%)
**Age (median in years)**	65	64	66
**Gender**			
- **Male**	117 (66.9%)	62 (66.7%)	46 (67.6%)
- **Female**	58 (33.1%)	31 (33.3%)	22 (32.4%)
**Plasma cell dyscrasia**			
- **MG**	74 (42.3%)	38 (40.9%)	32 (47.1%)
- **SMM**	83 (47.4%)	46 (49.4%)	29 (42.7%)
- **MM**	18 (10.3%)	9 (9.7%)	7 (10.3%)
**Amyloidogenic light chain**			
- **Lambda**	137 (78.3%)	72 (77.4%)	55 (80.9%)
- **Kappa**	37 (21.1%)	21 (22.6%)	13 (19.1%)
- **NOS**	1 (0.6%)	0	0
**PCBM (median)**	13.5%	10%	15%
**dFLC (median mg/l.)**	208.5	260.1	170
**NTproBNP (median pg/ml.)**	4346	4535	3005.5
**hsTnT (median ng/l.)**	61.5	64	49
**Proteinuria (median g/d.)**	1.4	1	3.81
**GFR (median ml/min/1.73qm)**	63	66	64.5
**Mayo stages 2004**			
- **Stage I**	20 (11.6%)	10 (10.8%)	10 (15.4%)
- **Stage II**	50 (29.1%)	24 (25.8%)	24 (36.9%)
- **Stage IIIa**	48 (27.9%)	33 (35.4%)	13 (20%)
- **Stage IIIb**	54 (31.4%)	26 (28%)	18 (27.7%)
- **N.a.**	3	0	3
**Renal stages**			
- **Stage I**	43 (35.8%)	24 (40%)	18 (34.6%)
- **Stage II**	50 (41.7%)	22 (36.6%)	24 (46.2%)
- **Stage III**	14 (11.7%)	7 (11.7%)	7 (13.4%)
- **Dialysis**	13 (10.8%)	7 (11.7%)	3 (5.8%)
- **N.a.**	3	1	0
**iFISH**	**pos/neg (% of total)**	**pos/neg (% of total)**	**pos/neg (% of total)**
- **Gain/amplification 1q21 (+ 1q21)**	32/118 (21.3%/78.7%)	15/68 (18.1%/81.9%)	11/42 (20.8%/79.2%)
- **t(11;14)**	91/70 (56.5%/43.5%)	51/40 (57%/43%)	35/21 (62.5%/37.5%)
- **Hyperdiploidy**	25/97 (20.5%/79.5%)	19/53 (26.4%/73.6%)	5/34 (12.8%/87.2%)
- **Deletion 13q14**	31/64 (32.6%/67.4%)	23/47 (32.9%/67.1%)	5/10 (33.3%/66.7%)
- **Deletion 16q23**	26/127 (17%/83%)	14/71 (16.5%/83.5%)	10/44 (18.5%/81.5%)
**Treatment response (4 weeks)**	**Number of patients (%)**	**Number of patients (%)**	**Number of patients (%)**
- **CR**	18 (17.1%)	13 (19.7%)	5 (12.8%)
- **VGPR/Low dFLC-PR**	22 (21%)	13 (19.7%)	9 (23.1%)
- **PR**	23 (21.9%)	16 (24.2%)	7 (18%)
- **NR**	31 (29.5%)	18 (27.3%)	13 (33.3%)
- **Deaths under treatment**	11 (10.5%)	6 (9.1%)	5 (12.8%)
- **Deaths before treatment**	12		
- **Naive patients**	2		
- **Not available data**	56		

*CR*: complete remission, *dFLC*: difference between involved to non-involved light-chain, *GFR*: glomerular filtration rate, *hsTnT*: high-sensitive troponin T,* iFISH*: fluorescence in situ hybridization, *MG*: monoclonal gammopathy, *MM*: multiple myeloma, *N.a*. not available data, *NOS*: not-other specified, *NR*: no response, *NTproBNP*: N-terminal prohormone of brain natriuretic peptide, *PCBM*: plasma cell infiltration in bone marrow,* PR*: partial remission, *SMM*: smoldering multiple myeloma. *VGPR*: very good partial remission

### Hematological response, hem-EFS and OS

To evaluate the hematological response to first line treatment, the total cohort was divided into two groups: patients treated in the daratumumab era, primarily with daratumumab-containing regimens (often in combination with cyclophosphamide bortezomib and dexamethasone) (93 patients, Table 1), and patients who were treated in the pre-daratumumab era, mostly with bortezomib-based regimens (68 patients, Table 1). In the later one, seven patients were treated with other regimens: five with melphalan and dexamethasone, one with high-dose melphalan and one with lenalidomide and dexamethasone.

Hematological response was assessed at 4 weeks as recommended by the International Society of Amyloidosis [30]. Adequate hematological response was defined as achieving either a complete response (CR) or a very good partial remission (VGPR) at 4 weeks after initiating 1st line treatment. Patients with dFLC between 20 and 50 mg/l were analyzed according Dittrich et al. [31].

Hem-EFS was defined as the time from diagnosis to death, hematological or organ progression according to consensus criteria or start of second line-treatment, whichever occurred first. OS was calculated from the date of diagnosis to death and censored for loss to follow-up or to the date of heart transplantation.

### Statistical analyses

Statistical analyses were conducted using the Mann-Whitney test to identify any significant differences among continuous disease variables related to iFISH aberrations, Fisher´s exact test for categorical variables and the log rank test for Kaplan-Meier survival analyses. The reversed Kaplan-Meier method was used to calculate the median follow-up time. Univariate and multivariate Cox regression models were performed to identify prognostic factors predictive for hematological response at 4 weeks, hem-EFS and OS stratified by treatment group (pre-daratumumab era and daratumumab era). Statistical significance was determined using GraphPad PRISM for Mac (Version 10.2.3) with *p*-values < 0.05 considered significant.

## Results

### Patient characteristics

The study included 175 consecutive patients with histologically confirmed AL amyloidosis who presented at our amyloidosis outpatient clinic. Median age at diagnosis was 65 years, 117 (67%) patients were male and 137 (78%) had an amyloidogenic light chain type lambda (Table 1). The underlying plasma cell disease was classified as monoclonal gammopathy (*n* = 74, 42.3%), smoldering multiple myeloma (*n* = 83, 47.4%), and multiple myeloma (*n* = 18, 10.3%). According to the modified Mayo 2004 staging system, 48 (27.9%) patients were in stage IIIa and 54 (31.4%) in stage IIIb (Table 1). Further details are shown in Table 1.

### Frequency of iFISH aberrations

**Fig. 1:**
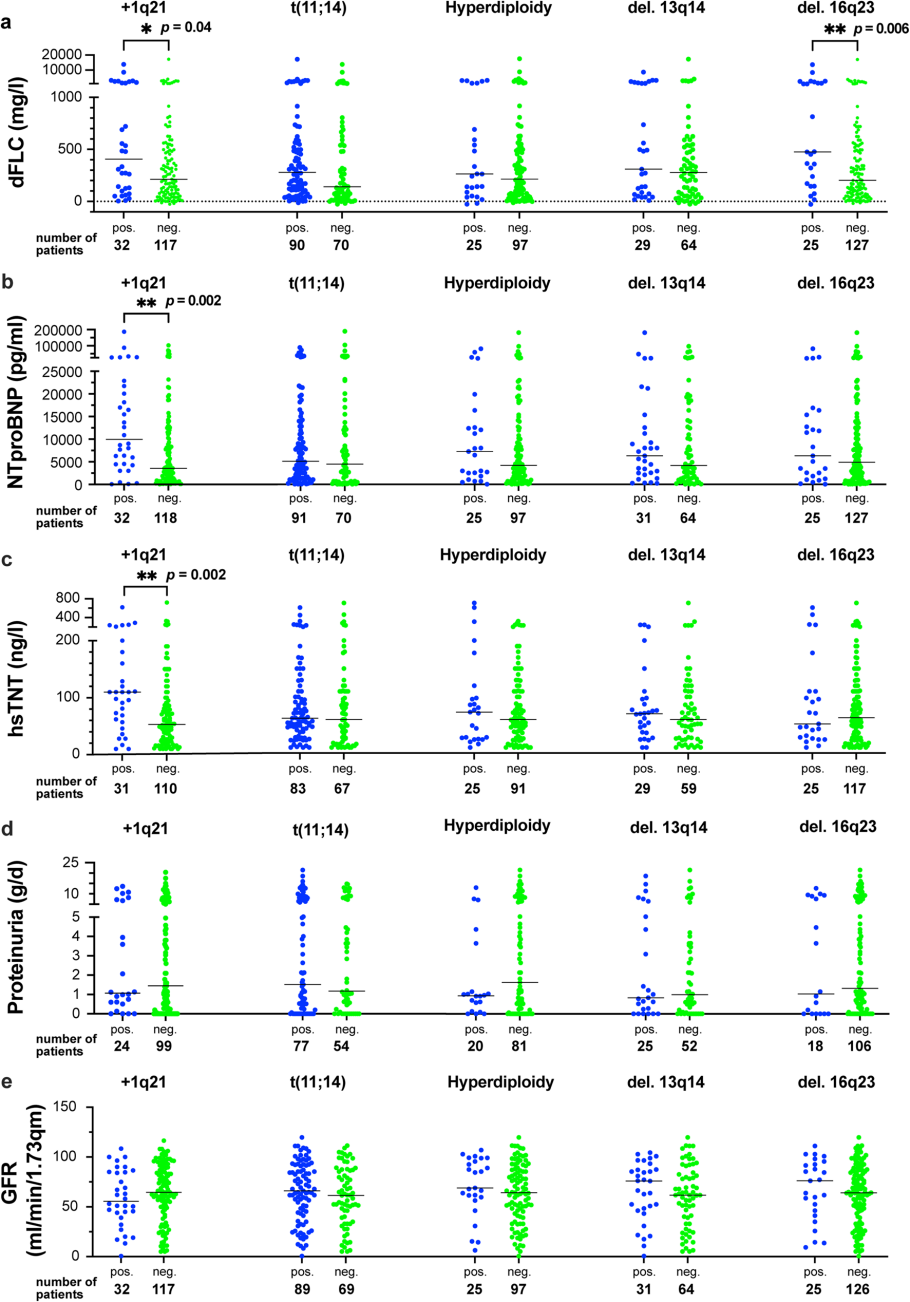
Levels of disease parameters in the most common five aberrations in AL amyloidosis. **a** dFLC: difference between involved and noninvolved light chain, **b** NTproBNP: N-terminal prohormone of brain natriuretic peptide, **c** hsTnT: highsensitive troponin T, **d** Proteinuria, **e** GFR: glomerular filtrations rate

In patients with available iFISH data, at least one aberration at diagnosis was detected in 93.1% of cases. The most commonly detected aberrations were t(11;14) in 57%, deletion 13q14 in 33%, +1q21 in 21%, hyperdiploidy in 21% and deletion 16q23 in 17% (Table 1). Frequencies of other, less common cytogenetic aberrations are shown in Supplementary Table 1. A Venn diagram illustrating the combinations of cytogenetic changes is presented in Supplementary, Fig. 1.

### Levels of disease parameters and Mayo stages regarding iFISH aberrations

**Fig. 2:**
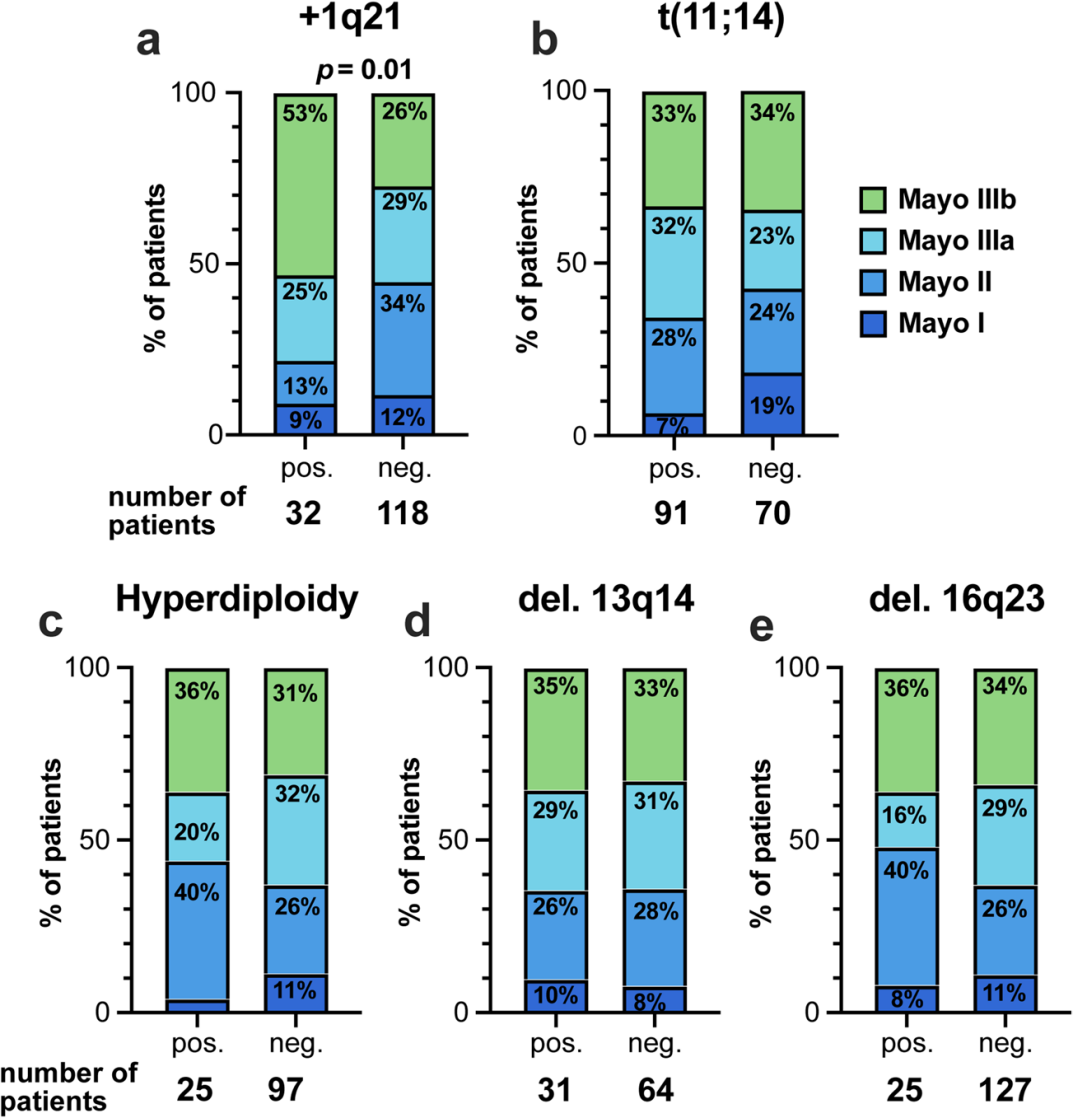
Distribution of Mayo stages in the most common five aberrations in AL amyloidosis. **a** +1q21, **b** t(11;14), **c** Hyperdiploidy, **d** del. 13q14, **e** del. 16q23. Neg: negative, pos: positive

To investigate the potential influence of individual iFISH aberrations on established hematological, cardiac and renal disease parameters, we compared the respective values based on the presence of specific cytogenetic aberrations. Interestingly, we observed significant elevations in dFLC levels in patients positive for + 1q21 (median 407 vs. 213 mg/l, *p* = 0.04), and deletion 16q23 (median 476 vs. 204 mg/l, *p* = 0.006), with a trend noted in t(11;14) (median 280 vs. 141, *p* = 0.08) (Fig. 1a). Of note, among these aberrations, only + 1q21 was significantly associated with increased levels of cardiac biomarkers, including N-terminal prohormone of brain natriuretic peptide (NTproBNP) (median 9 945 vs. 3 538 pg/ml, *p* = 0.002) and high-sensitive troponin T (hsTnT) (median 110 vs. 53 ng/l., *p* = 0.002) (Fig. 1b and c). Cytogenetic aberrations did not impact proteinuria or glomerular filtration rate (Fig. 1d and e). This resulted in a significantly increased proportion of patients with advanced Mayo stage IIIb who were positive for + 1q21 (53% vs. 26%, *p* = 0.01) (Fig. 2a), while no significant differences were observed in the distribution of Mayo stages between other positive and negative aberrations (Fig. 2b and e).

### Plasma cell disease regarding iFISH aberrations

**Fig. 3:**
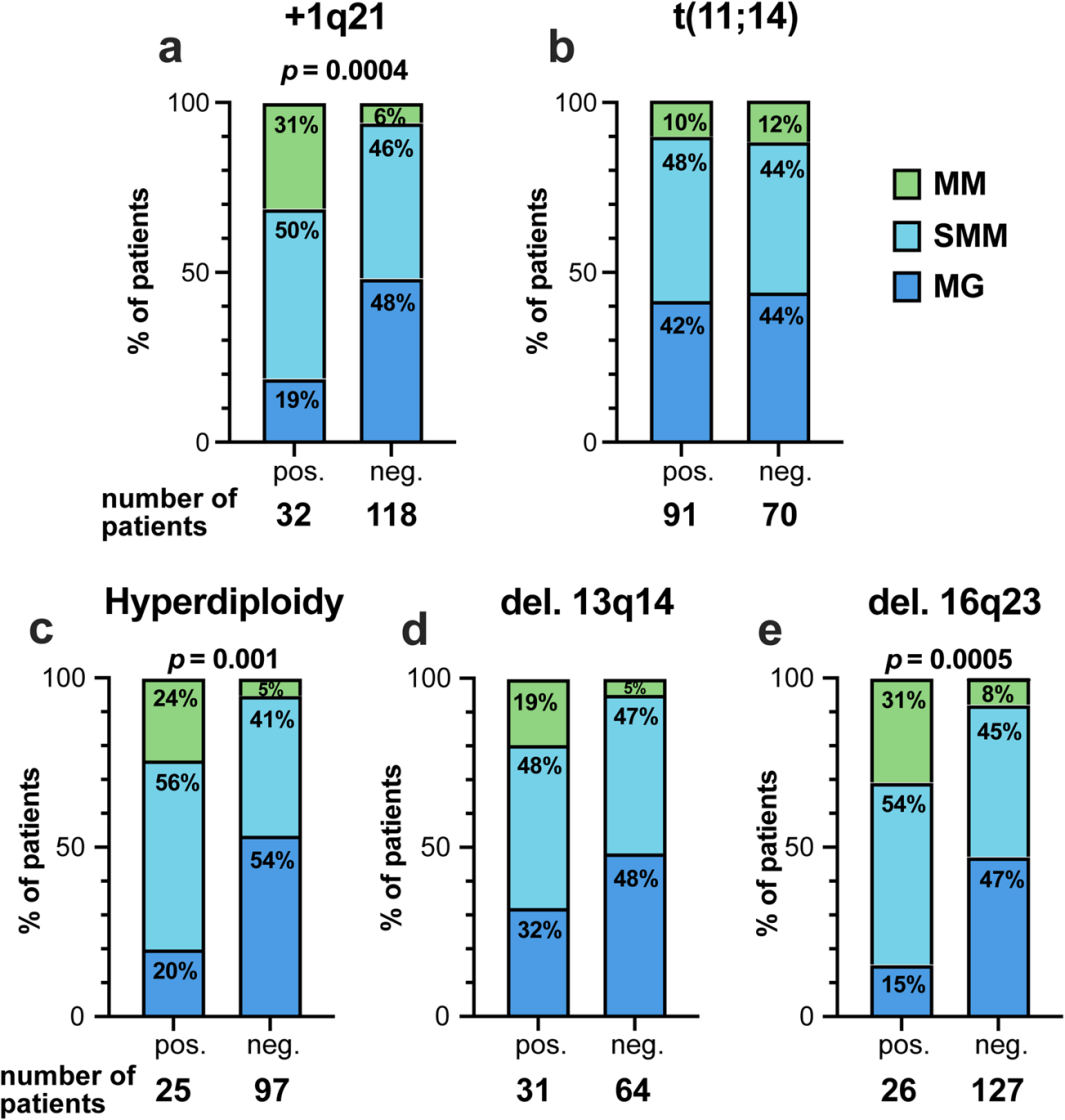
Distribution of plasma cell diseases in the most common five aberrations in AL amyloidosis. **a** +1q21, **b** t(11;14), **c** Hyperdiploidy, **d** del. 13q14, **e** del. 16q23. MG: monoclonal gammopathy, MM: multiple myeloma, neg: negative, pos: positive, SMM: smoldering multiple myeloma

When examining the distribution of underlying plasma cell diseases in relation to the cytogenetic aberrations, it became apparent that patients with + 1q21 had significantly more advanced disease; the proportion of patients with multiple myeloma was 31% vs. 6%, *p* = 0.0004 (Fig. 3a). To a lesser extent, we also found more advanced plasma cell disease in patients with deletion 16q23 and hyperdiploidy (Fig. 3c and e).

### Hematological response, hem-EFS and OS regarding iFISH aberrations and treatment era

**Fig. 4:**
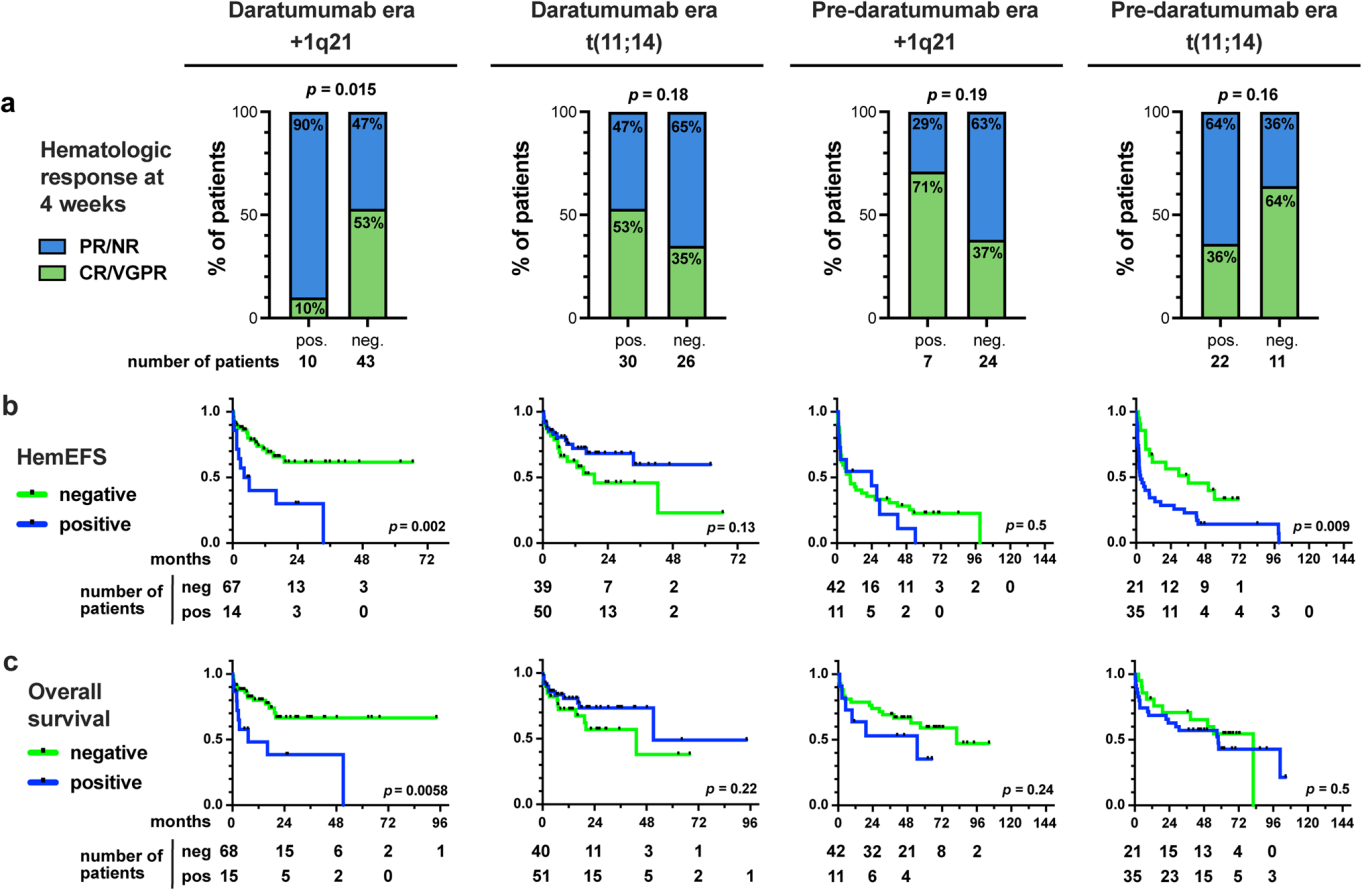
**a** Hematologic response at 4 weeks after treatment, **b** hematologic event-free survival (hem-EFS) and **c** overall survival (OS) in the daratumumab and pre-daratumumab era. Only patients with evaluable hematologic response data were included. Deaths were excluded from the analysis. CR: complete remission, NR: no response, PR: partial remission, VGPR: very good partial remission

To investigate the prognostic role of disease parameters and cytogenetic changes, predictive for hematological response at 4 weeks, hem-EFS and OS, univariate and multivariate Cox regression models as well as Kaplan Meyer survival analyses were performed (Tables 2 and 3; Fig. 4).

**Table 2:** Univariate and multivariate regression analysis for hematologic response at 4 weeks, hematologic event-free survival (hem-EFS) and overall survival (OS) in patients treated in the daratumumab era. Deaths were excluded from the hematologic response analysis

Variable	Hematologic response at 4 weeks CR/VGPR (control) vs. PR/NR			Hematologic event-free survival (hem-EFS)			Overall survival (OS)		
	*n*	HR (95%CI)	*p*-value	*n*	HR (95%CI)	*p*-value	*n*	HR (95%CI)	*p*-value
Age ≥ 65 years	57	1.016 (0.51–1.99)	0.96	91	1.71 (0.83–3.54)	0.14	93	1.67 (0.77–3.73)	0.19
dFLC ≥ 180 mg/L	57	1.85 (0.92–3.97)	0.09	91	3.18 (1.44–8.01)	**0.007**	93	2.65 (1.15–6.85)	**0.03**
PCBM ≥ 10%	57	1.83 (0.90–4.01)	0.11	91	1.48 (0.69–3.54)	0.34	93	1.88 (0.79–5.14)	0.17
MM Control: MG + SMM	57	2.92 (1.08–6.69)	**0.02**	91	2.74 (1.01–6.30)	**0.03**	93	3.13 (1.14–7.42)	**0.01**
Mayo IIIb	57	1.19 (0.56–2.40)	0.62	91	3.002 (1.47–6.11)	**0.0023**	93	3.01 (1.37–6.59)	**0.005**
NTproBNP ≥ 8500 pg/ml	57	1.29 (0.62–2.56)	0.48	91	3.35 (1.64–6.87)	**0.0008**	93	3.45 (1.58–7.63)	**0.002**
hsTnT ≥ 50 ng/l	55	1.11 (0.54–2.44)	0.79	88	1.91 (0.88–4.58)	0.12	90	1.83 (0.79–4.71)	0.18
GFR < 50 ml/min/1.73	56	0.88 (0.40–1.80)	0.74	90	1.51 (0.71–3.07)	0.26	92	1.57 (0.69–3.41)	0.26
PU ≥ 3.5 g/d	49	0.62 (0.23–1.46)	0.31	76	0.45 (0.15–1.13)	0.12	77	0.30 (0.07–0.89)	0.056
Gain/ampl. 1q21	53	3.52 (1.56–7.48)	**0.0015**	81	3.12 (1.39–6.61)	**0.003**	83	3.002 (1.26–6.67)	**0.009**
t(11;14)	56	0.51 (0.25–1.01)	0.056	89	0.58 (0.28–1.18)	0.13	91	0.62 (0.28–1.35)	0.23
Hyperdiploidy	46	2.01 (0.84–4.47)	0.09	70	1.74 (0.73–3.93)	0.19	72	2.32 (095-5.51)	0.056
Del. 13q14	46	1.47 (0.66–3.13)	0.33	68	0.58 (0.21–1.38)	0.24	70	0.75 (0.27–1.84)	0.54
Del. 16q23	54	1.40 (0.47–3.35)	0.48	83	0.92 (0.33–2.15)	0.85	85	1.15 (0.41–2.74)	0.77
**Multivariate analysis**									
dFLC ≥ 180 mg/L	53	2.05 (0.92–5.00)	0.09	81	1.81 (0.75–4.80)	0.20	83	1.51 (0.61–4.09)	0.38
MM	53	1.15 (0.34–3.71)	0.81	81	0.69 (0.21–2.15)	0.54	83	0.97 (0.27–3.31)	0.96
Mayo IIIb	53	0.87 (0.38–1.85)	0.72	81	2.1 (0.87–4.89)	0.09	83	2.01 (0.78–4.98)	0.14
Gain/ampl. 1q21	53	3.03 (1.04–7.63)	**0.03**	81	2.6 (0.97–6.35)	**0.04**	83	2.31 (0.78–6.00)	0.10

*CR*: complete remission, *dFLC*: difference between involved to non-involved light chain, *GFR*: glomerular filtration rate, *hsTnT*: high-sensitive troponin T, *MG*: monoclonal gammopathy, *MM*: multiple myeloma, *NR*: no response, *NTproBNP*: N-terminal prohormone of brain natriuretic peptide, *PCBM*: plasma cell infiltration in bone marrow, *PR*: partial remission, *PU*: proteinuria, *SMM*: smoldering multiple myeloma, *VGPR*: very good partial remission

**Table 3:** Univariate and multivariate regression analysis for hematologic response at 4 weeks, hem-EFS, and OS in patients treated in the pre-daratumumab era. Deaths were excluded from the hematologic response analysis

Variable	Hematological response at 4 weeks CR/VGPR (control) vs. PR/ NR			Hematologic event-free survival (hem-EFS)			Overall survival (OS)		
	*n*	HR (95%CI)	*p*-value	*n*	HR (95%CI)	*p*-value	*n*	HR (95%CI)	*p*-value
Age ≥ 65 years	34	1.02 (0.42–2.71)	0.96	68	1.48 (0.85–2.69)	0.18	68	3.08 (1.36–8.27)	**0.01**
dFLC ≥ 180 mg/L	37	2.42 (1.01–6.37)	0.054	67	2 (1.15–3.45)	**0.01**	67	1.10 (0.53–2.21)	0.79
PCBM ≥ 10%	36	0.67 (0.28–1.77)	0.39	65	1.26 (0.72–2.25)	0.43	65	0.87 (0.43–1.78)	0.69
MM Control: MG + SMM	37	1.51 (0.35–4.45)	0.50	68	3 (1.17–6.53)	**0.011**	68	2.74 (1.01–6.31)	**0.03**
Mayo IIIb	37	0.84 (0.30–2.03)	0.71	65	2.5 (1.34–4.52)	**0.003**	65	3.47 (1.63–7.24)	**0.001**
NTproBNP ≥ 8500 pg/ml	37	0.83 (0.30–2.03)	0.71	66	2.35 (1.29–4.15)	**0.004**	66	3.12 (1.51–6.33)	**0.002**
hsTnT ≥ 50 ng/l	36	0.74 (0.31–1.75)	0.48	62	2.2 (1.21–3.87)	**0.009**	62	3.49 (1.60–8.21)	**0.002**
GFR < 50 ml/min/1.73	37	2.56 (1.016–6.061)	**0.036**	66	1.8 (0.90–3.25)	0.08	66	1.73 (0.75–3.67)	0.17
PU ≥ 3.5 g/d	34	0.57 (0.21–1.39)	0.23	61	0.8 (0.44–1.39)	0.40	61	0.89 (0.43–1.89)	0.77
Gain/ampl. 1q21	31	0.36 (0.05–1.27)	0.17	53	1.3 (0.59–2.51)	0.51	53	1.73 (0.62–4.24)	0.25
t(11;14)	33	2.14 (0.76–7.58)	0.18	56	2.3 (1.23–4.64)	**0.01**	56	1.24 (0.58–2.81)	0.58
Hyperdiploidy	24	0.61 (0.09–2.27)	0.52	39	0.7 (0.21–1.82)	0.52	39	0.69 (0.11–2.41)	0.62
Del. 13q14	12	0.32 (0.05–1.41)	0.17	15	0.9 (0.23–2.65)	0.81	15	2.70 (0.62–11.84)	0.17
Del. 16q23	31	1.74 (0.61–4.49)	0.27	54	1.99 (0.92–3.96)	0.061	54	0.72 (0.21–1.89)	0.55
**Multivariate analysis**									
dFLC ≥ 180 mg/L	**-**	**-**	-	54	1.5 (0.79–2.96)	0.19	-	-	-
MM	-	-	-	54	3.6 (1.16–9.22)	**0.01**	65	2.11 (0.76–4.96)	0.11
Mayo IIIb	-	-	-	54	3.8 (1.86–7.83)	**0.0002**	65	3.20 (1.48–6.76)	**0.002**
t(11;14)	-	-	-	54	3.4 (1.64–7.29)	**0.001**	-	-	-

*CR*: complete remission, *dFLC*: difference between involved to non-involved light chain, *GFR*: glomerular filtration rate, *hsTnT*: high-sensitive troponin T, *MG*: monoclonal gammopathy, *MM*: multiple myeloma, *NR*: no response, *NTproBNP*: N-terminal prohormone of brain natriuretic peptide, *PCBM*: plasma cell infiltration in bone marrow, *PR*: partial remission, *PU*: proteinuria, *SMM*: smoldering multiple myeloma, *VGPR*: very good partial remission

The total cohort was divided into two groups based on treatment era: Patients primarily treated with daratumumab-containing regimens in the daratumumab era (93 patients, Table 2) and patients who were treated with other regimens particularly bortezomib-based regimens in the pre-daratumumab era (68 patients, Table 3). Patients who died before the start of planned therapy (*n* = 12) and patients who did not require therapy due to involvement of clinically irrelevant organs (*n* = 2) were not assigned to either treatment group.

In the daratumumab era and after a median follow-up time of 17 months, +1q21-positive patients had a worse hematological response at 4 weeks (only 10% CR or VGPR vs. 53%, *p* = 0.015) (Fig. 4a), a shorter hem-EFS (median 5.1 months vs. not reached, *p* = 0.002) and a worse OS (median 7.2 months vs. not reached, *p* = 0.0058) (Fig. 4b and c). In multivariate analyses, the presence of + 1q21 was independently associated with reduced hematological response at 4 weeks and poorer hem-EFS (Table 2). In contrast, there was no impact on hematological response, hem-EFS or OS in patients positive for t(11;14) (Fig. 4a-c).

In the pre-daratumumab era and after a median follow-up time of 63 months, t(11;14)-positive patients had a tendency for a reduced rate of hematological response (36% CR or VGPR vs. 64%, *p* = 0.16) and a shorter hem-EFS (median 2.9 months vs. 36.2 months, *p* = 0.009) with no effect on OS (Fig. 4a-c). In the multivariate analysis, the presence of t(11;14), multiple myeloma, and advanced Mayo stage IIIb were independently associated with a worse hem-EFS, while only Mayo stage IIIb was a significant negative predictor for OS (Table 3). In contrast, +1q21 did not affect hem-EFS or OS (Table 3; Fig. 4B and C).

## Discussion

In this study, we evaluated the role of the most common cytogenetic aberrations in systemic AL amyloidosis and their influence on disease activity parameters at diagnosis. We further stratified patients based on treatment era and identified negative predictive factors for hematological response at 4 weeks, hem-EFS, and OS.

The most frequently detected aberrations in our cohort were t(11;14), +1q21, hyperdiploidy, deletion 13q14 and deletion 16q23 (Table 1, Supplementary Table 1), in broad agreement with previous studies [14, 16, 17, 19, 23, 32]. The majority of patients had advanced Mayo 2004 stage III (59.3%) at diagnosis, with 31.4% having Mayo 2004 stage IIIb (Table 1). This relatively high proportion of severely ill patients reflects the central location of our center in one of the largest conurbations in Europe, where obstacles due to long journeys may play a subordinate role.

When analyzing the levels of disease parameters according to cytogenetic aberrations, we observed a significant elevation of dFLC levels in + 1q21-positive patients and those with deletion 16q23, while no differences were found in dFLC levels in the other analyzed cytogenetic aberrations (Fig. 1a). Only the presence of + 1q21 was associated with a significant increase in the levels of cardiac biomarkers NTproBNP and hsTNT (Fig. 1b and c), which was also reflected in the significantly higher proportion of patients with advanced Mayo 2004 stage IIIb (53 vs. 26%, *p* = 0.01). In contrast, there were no differences in the distribution of Mayo stages among the other aberrations (Fig. 2). Although the dFLC levels in patients with deletion 16q23 were higher than those in + 1q21-positive patients, this did not translate into higher levels of cardiac parameters or in an increased proportion of patients with advanced Mayo stage. This finding confirms previous studies [32] and supports the notion that increase in dFLC is not directly responsible for advanced Mayo 2004 stages.

Although the rate of advanced plasma cell disease was significantly higher in patients positive for + 1q21, hyperdiploidy and deletion 16q23, this factor did not impact hem-EFS, OS or hematological response in the daratumumab era. In contrast, multiple myeloma was a significant negative predictor for hem-EFS in the pre-daratumumab era (Tables 2 and 3; Fig. 3).

Furthermore, we analyzed various factors that could predict hematological response at 4 weeks, hem-EFS and OS in both treatment eras using univariate and multivariate Cox regression analyses. The presence of + 1q21 was an independent negative prognostic factor for hematological response at 4 weeks and hem-EFS in patients treated with daratumumab-containing regimens in first-line treatment (Table 2), which confirms recently published findings and is in line with studies in relapse [22, 33]. As shown in the Andromeda study [10, 21], we no longer found a negative prognostic effect of t(11;14) in patients treated in the daratumumab era. In the pre-daratumumab era, the presence of t(11;14), advanced plasma cell disease and advanced Mayo stage were independent negative prognostic factors for shorter hem-EFS, whereas + 1q21 did not play a significant role even in the univariate analysis (Table 3). This is consistent with the published literature [17, 19, 20]. We could not find a negative effect of t(11;14) on OS, possibly because patients in later lines were treated with daratumumab.

Patients positive for + 1q21 benefited less from the treatment with daratumumab than 1q21-negative patients (Fig. 4a; Table 2). While the rate of CR or VGPR at 4 weeks in 1q21-negative patients improved from 38% in the pre-daratumumab era to 52% in the daratumumab era, it declined from 71 to 10% in + 1q21 patients. Of note, in the daratumumab era, bortezomib was added in later treatment cycles in some patients with advanced Mayo stage III, in line with the EMN 22 study [34]. The concept of initially administering daratumumab therapy to patients with cardiac Mayo Stage IIIb and adding further components at later stages has shown to be favorable in retrospective observations, most likely because this approach reduces toxicity [35]. This treatment reduction may in addition have led to poorer 4-week hematological response due to the increased proportion of patients with advanced Mayo stages in + 1q21 patients. Nevertheless, our data clearly indicate that the subpopulation of + 1q21 positive patients may initially be insufficiently treated with this approach.

In conclusion, the present study shows for the first time that + 1q21-positive patients are significantly more likely to present with advanced heart disease at diagnosis, as well as more advanced plasma cell disease, which already gives patients a poorer prognosis. In addition, in the context of daratumumab-containing regimens, +1q21 is an independent negative prognostic parameter, affecting hematological response at 4 weeks and hem-EFS. Overall survival in + 1q21 treated with daratumumab containing regimens is dramatically worse, alternative therapeutic approaches such as CAR-T therapies or bispecific antibodies should be further investigated in this doubly disadvantaged group.

## Supplementary Information

Below is the link to the electronic supplementary material. 

ESM1 (DOCX 69.0 KB)

## Data Availability

The data that support the findings of this study are available on request from the corresponding author. The data are not publicly available due to restrictions e.g. their containing information that could compromise the privacy of research participants.

## Abbreviations

+ 1q21: gain or amplification of 1q21
AL: light chain amyloidosis
CR: complete response
iFISH: interphase fluorescence in situ hybridization
hem-EFS: hematological event-free survival
hsTnT: high-sensitive troponin T
NTproBNP: N-terminal prohormone of brain natriuretic peptide
OS: overall survival
VGPR: very good partial remission
