# Supplementary material for: Gain or amplification of 1q21 in systemic light chain amyloidosis is associated with advanced Mayo stage, plasma cell disease and worse overall survival
Source: Ann Hematol. 2025 Mar 22;104(3):1777–88. doi: 10.1007/s00277-025-06256-7 (PMC12031875; doi:10.1007/s00277-025-06256-7)
Supplement: Supplementary file 1 — (DOCX 69.0 KB) [file 277_2025_6256_MOESM1_ESM.docx]

**Supplementary**

**Table 1.**

| **iFISH-aberrations** | **Number of patients (%)** |
| --- | --- |
| **Translocations** | |
| **t(11;14)** |  |
| positive | 91 (56.5%) |
| negative | 70 (43.5%) |
|  |  |
| **t(4;14)** |  |
| positive | 1 (0.7) |
| negative | 147 (99.3%) |
|  |  |
| **t(14;16)** |  |
| positive | 6 (4.1%) |
| negative | 141 (95.9%) |
|  |  |
| **t(14;20)** |  |
| positive | 4 (3%) |
| negative | 129 (97%) |
|  |  |
| **Deletions** | |
| **Deletion 17p13** |  |
| positive | 3 (1.9%) |
| negative | 153 (98.1%) |
|  |  |
| **Deletion 16q23** |  |
| positive | 26 (17%) |
| negative | 127 (83%) |
|  |  |
| **Deletion 13q14** |  |
| positive | 31 (32.6%) |
| negative | 64 (67.4%) |
|  |  |
| **Gains and hyperdiploidy** | |
| **Gain/amplification 1q21 (+1q21)** |  |
| positive | 32 (21.3%) |
| negative | 118 (78.7%) |
|  |  |
| **Hyperdiploidy*** |  |
| positive | 25 (20.5%) |
| negative | 97 (79.5%) |
|  |  |
| **5p15 ploidy** |  |
| positive | 19 (16.2%) |
| negative | 98 (83.8%) |
|  |  |
| **9q22 ploidy** |  |
| positive | 27 (23.3%) |
| negative | 89 (76.7%) |
|  |  |
| **15q22 ploidy** |  |
| positive | 26 (21.3%) |
| negative | 96 (78.7%) |

**Table 1. The frequency of Interphase fluorescence in situ hybridization (iFISH) aberrations in patients with AL amyloidosis.** *Hyperdiploidy was defined as detection of at least 2 from the 3 aberrations +5p15, +9q22, and +15q22 in at least 10% of the nuclei.

**Fig. 1**

**Fig. 1** Venn-diagram viewing combinations of different aberrations. Only patients with available data regarding all the five most common aberrations together were included.
